# Meningitis associated with strongyloidiasis in an area endemic for strongyloidiasis and human T-lymphotropic virus-1: a single-center experience in Japan between 1990 and 2010

**DOI:** 10.1007/s15010-013-0483-2

**Published:** 2013-06-13

**Authors:** Y. Sasaki, T. Taniguchi, M. Kinjo, R. L. McGill, A. T. McGill, S. Tsuha, S. Shiiki

**Affiliations:** 1 Department of Medicine, Okinawa Chubu Hospital, Okinawa, Japan; 2 Division of Infectious Diseases, Okinawa Chubu Hospital, Okinawa, Japan; 3 Department of Medicine, Allegheny General Hospital, Pittsburgh, PA USA; 4 Division of Infectious Diseases, Butler Memorial Hospital, Butler, PA USA; 5 Present Address: Department of Medicine, Okinawa Yaeyama Hospital, 732 Okawa, Ishigaki, Okinawa 907-0022 Japan

**Keywords:** Strongyloidiasis, *Streptococcus bovis*, Meningitis, Culture-negative meningitis, Human T-lymphotropic virus-1

## Abstract

Meningitis caused by enteric flora is a known complication of strongyloidiasis, and human T-lymphotropic virus-1 (HTLV-1) predisposes individuals to severe strongyloidiasis. We reviewed the clinical features of bacterial meningitis associated with strongyloidiasis seen at a single center in subtropical Japan, in an area endemic for both strongyloidiasis and HTLV-1. We found 33 episodes in 21 patients between 1990 and 2010. The results were remarkable for the high incidence of meningitis due to Gram-positive cocci (27.3 %), especially *Streptococcus bovis*, and culture-negative cases (42.4 %). Given the high incidence of Gram-positive meningitis, a modified approach to corticosteroid use would be advisable in areas where strongyloidiasis is endemic, due to the potentially adverse consequences of glucocorticoid therapy.

## Introduction

*Strongyloides stercoralis* (*S. stercoralis*) is a nematode helminth that causes strongyloidiasis. It affects nearly 100 million people in tropical and subtropical areas of Europe, Africa, Latin America, and Asia. Most cases are chronic and asymptomatic, but severe infections such as hyperinfection syndrome (HIS) and disseminated strongyloidiasis (DS) occur when cellular immunity is compromised. In HIS (1.5–2.5 % of cases), manifestations are confined to the respiratory and gastrointestinal (GI) systems. DS, infection with multiple organ involvement other than the respiratory and GI systems such as the brain or the skin, may have a mortality of up to 87 % [1].

Bacterial meningitis caused by enteric flora is a potentially fatal complication of DS/HIS. Localized strongyloidiasis, as well as DS/HIS, can predispose individuals to bacterial meningitis with enteric organisms such as *Klebsiella pneumoniae* (*K. pneumoniae*), *Escherichia coli* (*E. coli*), and *Enterococcus* spp. in the absence of evidence for strongyloidiasis outside the GI tract [2, 3].

Human T-lymphotropic virus-1 (HTLV-1) is a retrovirus that infects 10–20 million people worldwide. The virus is highly prevalent in Japan, sub-Saharan Africa, the Caribbean, and South America. Southern Japan is the area with the highest HTLV-1 prevalence, with over 10 % of the general population being infected [4]. HTLV-1 can be transmitted from mother to child, through sexual contact, and through contaminated blood products. Most cases are asymptomatic, but adult T cell leukemia/lymphoma (ATLL) and HTLV-1-associated myelopathy/tropical spastic paresis may occur.

Co-infection with *S. stercoralis* and HTLV-1 predisposes individuals to DS/HIS, possibly because HTLV-1 interferes with Th2 cell responses to *S. stercoralis*, including eosinophil activation and production of interleukins and IgE [5]. Glucocorticoids, malignancies, human immunodeficiency virus (HIV), organ or marrow transplantation, hypogammaglobulinemia, leprosy, tuberculosis, and malnutrition also increase the risk [1]. This report delineates the clinical features of bacterial meningitis associated with strongyloidiasis seen at a single center in subtropical Japan, in an area endemic for both strongyloidiasis and HTLV-1.

## Methods

Cases with both bacterial meningitis and strongyloidiasis were extracted from a review of the discharge diagnoses for all hospitalizations at Okinawa Chubu Hospital between January 1990 and May 2010. This large general hospital serves a subtropical area. The prevalence of strongyloidiasis and HTLV-1 in this area is reportedly 6.3 and 14 %, respectively [5]. About 39,000 patients visit the emergency department and nearly 14,000 patients are hospitalized per year.

After review, we included cases that met the following inclusion criteria: (1) typical signs and symptoms of meningitis (headache, temperature >38 °C, nuchal rigidity, and/or altered consciousness); (2) cerebrospinal fluid (CSF) findings consistent with bacterial meningitis, including: neutrophil (PMN) predominance, low glucose, high protein, high opening pressures, organisms on Gram stain, or recovery of microorganisms from CSF or blood; (3) proof of coexistent strongyloidiasis (larvae or eggs from any specimens). Two cases in two patients were excluded during this process.

In addition to the inclusion criteria, we collected the following data: gender, age, outcome, symptoms, CSF findings, *S. stercoralis* results, HIV testing results, medical/social history, glucocorticoid use, colonoscopy results, and use of antibiotics and antihelminthics.

We performed a Mann–Whitney *U*-test for CSF statistical analysis and a one-sided Fisher’s exact test for outcome statistical analysis, using Stata software (version 12.1; StataCorp, College Station, TX, USA).

## Results

Thirty-three episodes in 21 patients were included in this report. The median age at diagnosis was 58 years and 11/21 (52.4 %) patients were male.

Table 1 shows the gender, age, culture results for CSF and blood, results of CSF Gram staining, serological results for HTLV-1, specimens from which *S. stercoralis* was detected, past history, initial antibiotics and antihelminthics (antibiotics were subsequently chosen according to culture results), and outcome of the patients. Six episodes of acute meningitis without proven concurrent strongyloidiasis were included because each of these patients had previous or subsequent episodes of proven strongyloidiasis associated with meningitis. Five of 21 patients (23.8 %) had more than one episode of meningitis. Of these five patients, four were treated with thiabendazole, and one was not treated during the first episode because he was not diagnosed with strongyloidiasis. Among the eight cases treated with ivermectin, none developed recurrent episodes of either strongyloidiasis or meningitis.

**Table 1:**
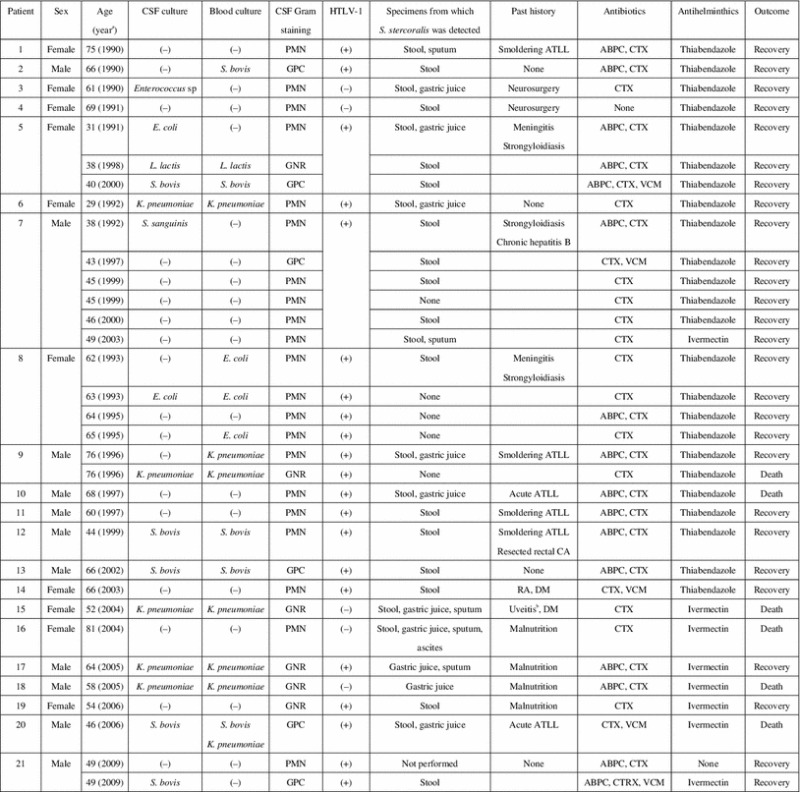
Clinical features of all episodes

*ABPC* ampicillin, *ATLL* adult T cell leukemia/lymphoma, *CA* carcinoma, *CTRX* ceftriaxone, *CTX* cefotaxime, *DM* diabetes mellitus, *E. coli* *Escherichia coli*, *GNR* Gram-negative rods, *GPC* Gram-positive cocci, *K. pneumoniae* *Klebsiella pneumoniae*, *L. lactic* *Lactococcus lactis*, *PMN* polymorphic nuclear neutrophils, *RA* rheumatoid arthritis, *S. bovis* *Streptococcus bovis*, *S. sanguinis* *Streptococcus sanguinis*, *VCM* vancomycin

^a^Indicates the year when diagnosis was made

^b^Oral glucocorticoid was prescribed

Table 2 summarizes the culture results for CSF and blood. *K. pneumoniae* was the most frequent pathogen, followed by *Streptococcus bovis* (*S. bovis*) and *E. coli*. No organism was isolated from CSF or blood cultures in 14/33 (42.4 %) episodes. Among culture-negative cases, only patient 20 was prescribed antibiotics prior to lumbar puncture. Gram-positive cocci (GPC) were detected in CSF and/or blood cultures in 9/33 (27.3 %) episodes.

**Table 2:** Summary of culture results

Organism	CSF culture (%)	Blood culture (%)
*K. pneumoniae*	5 (15.2)	7 (21.2)
*S. bovis*	5 (15.2)	5 (15.2)
*E. coli*	2 (6.1)	3 (9.1)
*L. lactis*	1 (3.0)	1 (3.0)
*Enterococcus* spp.	1 (3.0)	0
*S. sanguinis*	1 (3.0)	0
Negative	18 (54.5)	18 (54.5)

One case of mixed infection with *K. pneumoniae* and *S. bovis* is included in the blood culture results

*K. pneumoniae* *Klebsiella pneumoniae*, *S. bovis* *Streptococcus bovis*, *E. coli* *Escherichia coli*, *L. lactis* *Lactococcus lactis*, *S. sanguinis* *Streptococcus sanguinis*

Serology for HTLV-1 was positive in 16/21 (76.2 %) patients, including two cases of acute ATLL and four cases with smoldering ATLL. Among five cases without HTLV-1, two had previous neurosurgeries, two were diagnosed with malnutrition, and one received glucocorticoid for uveitis. Three patients had negative HIV test results (patients 6, 13, and 17), but no other patients were examined for HIV due to the low prevalence in Japan.

Twenty-seven of 33 cases (81.8 %) improved without neurological complications and 6/33 episodes (18.2 %) ended in fatalities. Two patients simultaneously developed acute ATLL. Among nine cases of GPC meningitis, only patient 21 received three doses of dexamethasone, 0.15 mg/kg every 8 h, which was discontinued after strongyloidiasis was diagnosed. All patients with GPC meningitis recovered without neurological complications, except for patient 20, who developed acute ATLL and died. There was no significant difference in outcome between culture-positive cases and culture-negative cases. Fatal cases comprised 4/19 (21.0 %) in culture-positive cases and 2/14 (14.2 %) in culture-negative cases (*p*-value 0.490).

Among 33 episodes of meningitis, fever was seen in 31 (93.9 %), headache in 30 (90.9 %), nausea and vomiting in 27 (81.8 %), rigors in 17 (51.5 %), and altered mental status in 15 (45.5 %).

*S. stercoralis* was detected in the stool in 26/33 (78.8 %) episodes, in gastric juice in 10/33 (30.3 %) episodes, in sputum in 5/33 (12.1 %) episodes, and in ascites in 1/33 (3.0 %) episodes. *S. stercoralis* was detected only in the GI tract specimens in 23/33 (69.7 %) cases. Only two patients underwent colonoscopy; patient 13 had diverticulosis and patient 21 had benign adenoma, both of whom had *S. bovis* meningitis. Nineteen of 21 (90.5 %) patients, including one patient with previously resected colon cancer, did not undergo colonoscopy.

Medians and interquartile ranges of CSF cell count, PMN percentage, sugar, CSF/blood sugar ratio, and protein were 1,440/mm^3^ (540–2,500), 84 % (71–96), 48 mg/dl (25–64), 39 % (12–53), and 117 mg/dl (84–237), respectively. There were no significant differences between culture-positive and culture-negative cases.

## Discussion

We present a series of 21 patients with neutrophil-dominant meningitis associated with strongyloidiasis, including 14 cases with negative CSF and blood cultures. *S. bovis* was the etiology for six patients, all of whom were positive for HTLV-1. The following important points emerged from our observations.

*S. bovis* is a GPC in the gut flora of 10–16 % of healthy individuals [6]. It may cause bacteremia, endocarditis, and meningitis in patients with GI lesions or colon cancer, and in neonates [7, 8]. *S. bovis* has been reported as a rare cause of bacterial meningitis associated with strongyloidiasis. Only four cases of bacterial meningitis due to *S. bovis* have been reported with strongyloidiasis; three had defective cellular immunity (two with HIV and one due to steroids) and one was reportedly immunocompetent [6, 9–11]. We detected six cases in a single center over 20 years, which suggests that the incidence of *S. bovis* meningitis in patients with strongyloidiasis may be higher than previously suspected. This interaction could impact the management of meningitis in geographic areas where HTLV-1 and strongyloidiasis are endemic.

The Practice Guidelines for the Management of Bacterial Meningitis published by the Infectious Diseases Society of America (IDSA) recommend dexamethasone for adults with suspected pneumococcal meningitis, with dosage continued if the CSF Gram stain shows Gram-positive diplococci [12]. This approach risks exacerbating strongyloidiasis in cases of meningitis due to GPC associated with strongyloidiasis, which cannot be reliably distinguished from pneumococci on Gram stain.

Glucocorticoid use is the strongest known risk factor for disseminated strongyloidiasis, due to the acquired defect in cell-mediated immunity. Glucocorticoids may act as ecdysteroids that accelerate the conversion of larvae from the rhabditiform state to the invasive filariform state [1, 13]. One case of DS was reported after only a single dose of dexamethasone prior to stereotactic radiosurgery [14]. Distinguishing pneumococcal meningitis from meningitis due to other GPC on clinical grounds or Gram stain alone is problematic. A modified approach to corticosteroid use in GPC meningitis would, therefore, be advisable in areas where strongyloidiasis is endemic. Patients with non-pneumococcal meningitis do not benefit from steroids, which are given to mitigate the harmful CSF inflammatory response. Patients with underlying immunologic diseases are less likely to mount self-damaging inflammatory responses. The good clinical outcomes seen in our cases, which were managed without glucocorticoids, may be attributable to these factors. A comprehensive microbiology approach using expanded techniques such as rapid latex agglutination testing may be helpful for the early identification of patients that can safely receive glucocorticoids, if this testing is locally available [12].

Of our cases, 42.4 % had negative cultures of both blood and CSF despite clinical meningitis and CSF pleocytosis, which were clinically indistinguishable from culture-positive cases. The postulated mechanism by which strongyloidiasis causes bacteremia and meningitis is that parasitic ulceration/perforations of the intestinal mucosa create a portal of entry for enteric bacteria to reach the bloodstream. Mantovani et al. [15] suggested that the bacteria are co-transported with the migrating parasites. Kishaba et al. [16] proposed an entity of “culture-negative suppurative meningitis” associated with DS. This hypothesis assumes that the parasite invades the meninges without any attending bacteria, and that *S. stercoralis* alone induces the intense inflammatory changes that mimic the CSF findings of bacterial meningitis. Given that the sensitivity of CSF or blood cultures in patients with bacterial meningitis is reportedly >70 % [17–19], our frequent observation of culture-negative suppurative meningitis lends support to this hypothesis.

Among 14 patients treated with thiabendazole for *S. stercoralis*, four (28.6 %) developed recurrence after treatment; no patient treated with ivermectin relapsed. These facts suggest that ivermectin may eradicate *S. stercoralis* more effectively than thiabendazole, in which case ivermectin should be considered for patients with underlying immunodeficiency or associated meningitis.

The fact that *S. stercoralis* was detected only in GI tract specimens in 69.7 % of cases supports the previously suggested theory that localized strongyloidiasis can cause enteric meningitis [2].

Limitations of this retrospective report of a single-center experience include the low percentage of cases in which colonoscopy excluded an additional portal of entry, such as structural lesions/cancers, and the infrequency of HIV testing. However, during the long-term follow-up of our geographically restricted cases, neither colon cancer nor HIV was diagnosed in any patient. Another limitation is a lack of information regarding the *S. bovis* speciation (*Streptococcus gallolyticus* or *Streptococcus infantarius*). Further observation of the association between these three infectious entities in additional institutions and other geographical areas will help establish the generalizability and importance of these observations.

## Conclusion

In areas where strongyloidiasis and human T-lymphotropic virus-1 (HTLV-1) are endemic, clinical meningitis is surprisingly common and frequently recurrent. Culture-negative meningitis and *Streptococcus bovis* meningitis may represent a larger proportion of cases than the meningitis seen in non-tropical areas. Clinicians should be aware that these cases may represent a special scenario in which the conventional use of glucocorticoids can pose a previously unrecognized hazard. Further study is needed in order to clarify the mechanisms by which these infections interact, and to define the prevalence and significance of accompanying structural colonic lesions.
